# A Puzzling Diagnosis of Cerebral Vein Thrombosis in a COVID-19-Vaccinated Patient

**DOI:** 10.7759/cureus.25860

**Published:** 2022-06-11

**Authors:** Isaac Alsallamin, Francisco J Somoza-Cano, Lara Zakarna, Pearl Aggarwal, Rusina Karia, Ameed Bawwab, Deema Chakhachiro, Afnan Alsallamin

**Affiliations:** 1 Internal Medicine, Northeast Ohio Medical University, Rootstown, USA; 2 Internal Medicine, St. Vincent Charity Medical Center, Cleveland, USA

**Keywords:** covid-19 vaccine side-effects, high flow nasal cannula (hfnc), ards (acute respiratory distress syndrome), high altitude pulmonary edema, covid-19 respiratory failure, covid-19 vaccine, covid19, cerebral vein thrombosis (cvt), stroke

## Abstract

Cerebral vein thrombosis (CVT) is a rare condition equivalent to deep vein thrombosis of the intracranial veins. Delayed diagnosis will result in severe and disabling complications. We report a case of a 59-year-old man with CVT with no significant past medical or surgical history. On admission, he reported right-sided numbness and weakness concerns, preceded by the sudden onset of bilateral vision loss and dysarthria. Magnetic resonance imaging and computed tomography scans confirmed the diagnosis of CVT. The most interesting relative risk factor was flying overseas twice a month for the last 10 years; each flight was longer than eight hours. Another possible contributing factor to our patient's condition was polycythemia, with a hemoglobin level of 19, but the most questionable and puzzling is the recent coronavirus disease 2019 (COVID-19) vaccination two months, eight months, and one year prior to admission. Our case highlights a rare COVID-19 vaccine-related CVT diagnosis and that close monitoring for new symptoms and signs is vital to prevent life-threatening complications, herniation, and hemorrhagic transformation.

## Introduction

Cerebral vein thrombosis (CVT) is the formation of clots in the brain venous system that block venous drainage, which can increase intracranial pressure, bleeding, or venous infarction. CVT is similar to deep vein thrombosis; the only difference being CVT occurs in the brain. The overall incidence of CVT is 11.6 per one million inhabitants, with a sex-specific rate of 15.1 per million female patients and 7.8 per million male patients [1]. Presentations range from nonspecific neurologic symptoms and signs like recurrent headache and nausea to abnormal sensory or motor deficits resembling brain stroke or transient ischemic stroke and minor stroke [2]. Traditional Virchow's triad can explain the pathophysiology and risk factors for CVT, stasis, vessel wall injury, and procoagulant status. Some are acquired and adjustable, such as surgery, pregnancy, and hormones; others are genetic, such as thrombophilia and polycythemia. However, in most cases, CVT is multifactorial [3]. Infection by the severe acute respiratory syndrome coronavirus 2 (SARS-CoV-2), the cause of coronavirus disease 2019 (COVID-19), is associated with arterial and venous thrombosis, a well-known phenomenon. The known mechanisms include endothelial dysfunction, inflammation, cytokine release, acute phase reactant proteins, activation of coagulopathy, and platelet dysregulation due to SARS-CoV-2 [4]. The COVID-19 vaccine induces venous thrombosis along the same pathways as the virus, as identified in several cases reported worldwide. More cases of venous thrombosis are connected to adenovirus-based vaccines than other vaccines (e.g., protein or messenger ribonucleic acid (mRNA)-based vaccines). Recent data suggest an excess event rate of CVT of 2.5 per 100,000 ChAdOx1 (AstraZeneca) recipients in the United States. Seventeen recipients of Ad26.COV2.S (Johnson & Johnson/Janssen) suffered from thrombosis and thrombocytopenia, with 14 cases of CVT; three cases were fatal, leading the Centers for Disease Control and the European Medicines Agency to recommend temporary discontinuation of this vaccine. Reported cases of vaccine-induced thrombotic thrombocytopenic showed the time from vaccine to symptoms onset ranged from five to 16 days following the ChAdOx1 vaccine [5,6]. We describe a case of a 59-year-old man with CVT with no significant past medical or surgical history following his third dose of the COVID-19 vaccine.

## Case presentation

A 59-year-old right-handed white man presented to the hospital with a sudden onset headache, right eye vision loss, and dysarthria. His past medical history was unremarkable. The patient reported that for the past 10 years, he had traveled overseas on flights lasting longer than eight hours twice a month. He received the COVID-19 vaccine two months, eight months, and one year before admission. A more detailed history found that for the past two weeks, he has had recurrent mild headaches that were sharp, not radiated, and rated at 6/10 on the visual analog pain scale. The headaches were felt primarily on the right side associated with right facial numbness, and they spontaneously resolved in less than one hour. He denied aggravating factors. On the day of admission, he reported right lower facial numbness, sudden painless right eye vision loss, dysarthria, and right upper extremity numbness and weakness that lasted for a few hours until he arrived at the hospital. He denied photophobia, nausea, or vomiting. There were no alleviating or aggravating factors, and he was resting at his home. His physical examination in the emergency department was positive for mild weakness on the right upper extremity (grade 4/5). Other findings were unremarkable. His blood pressure was 140/85 mmHg, his heart rate was 63 beats/minute, his temperature was 36.6°C, and his oxygen saturation was 99% on room air. He had polycythemia, with a hemoglobin level of 19 g/dL. His other initial laboratory results were within reference ranges.

**Figure 1:**
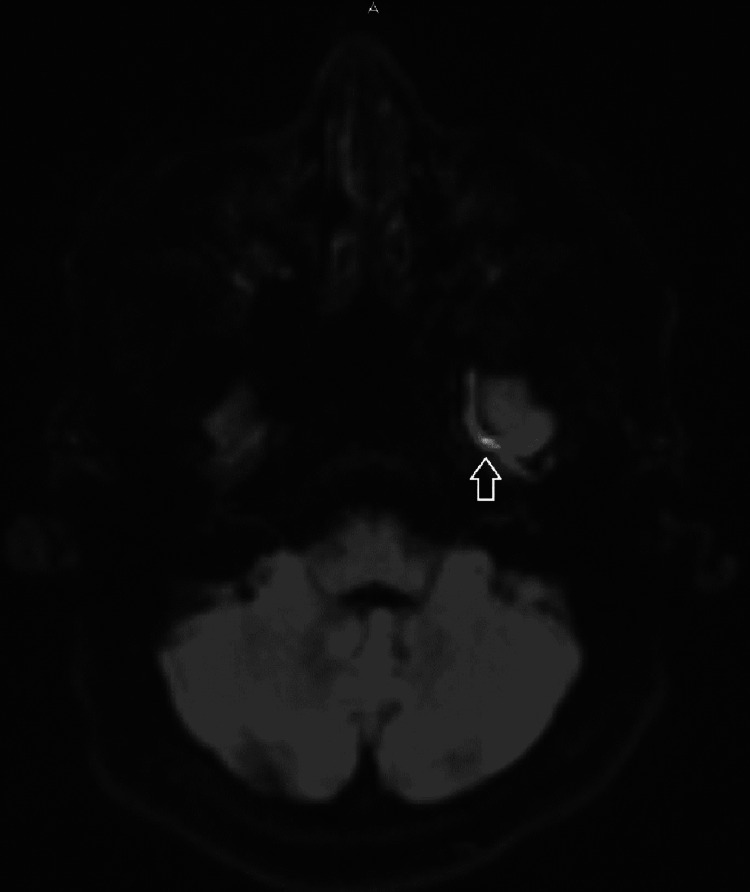
MRI-FLAIR with hyperintensity signal due to cerebral vein thrombosis (arrow) MRI: magnetic resonance imaging; FLAIR: fluid-attenuated inversion recovery

**Figure 2:**
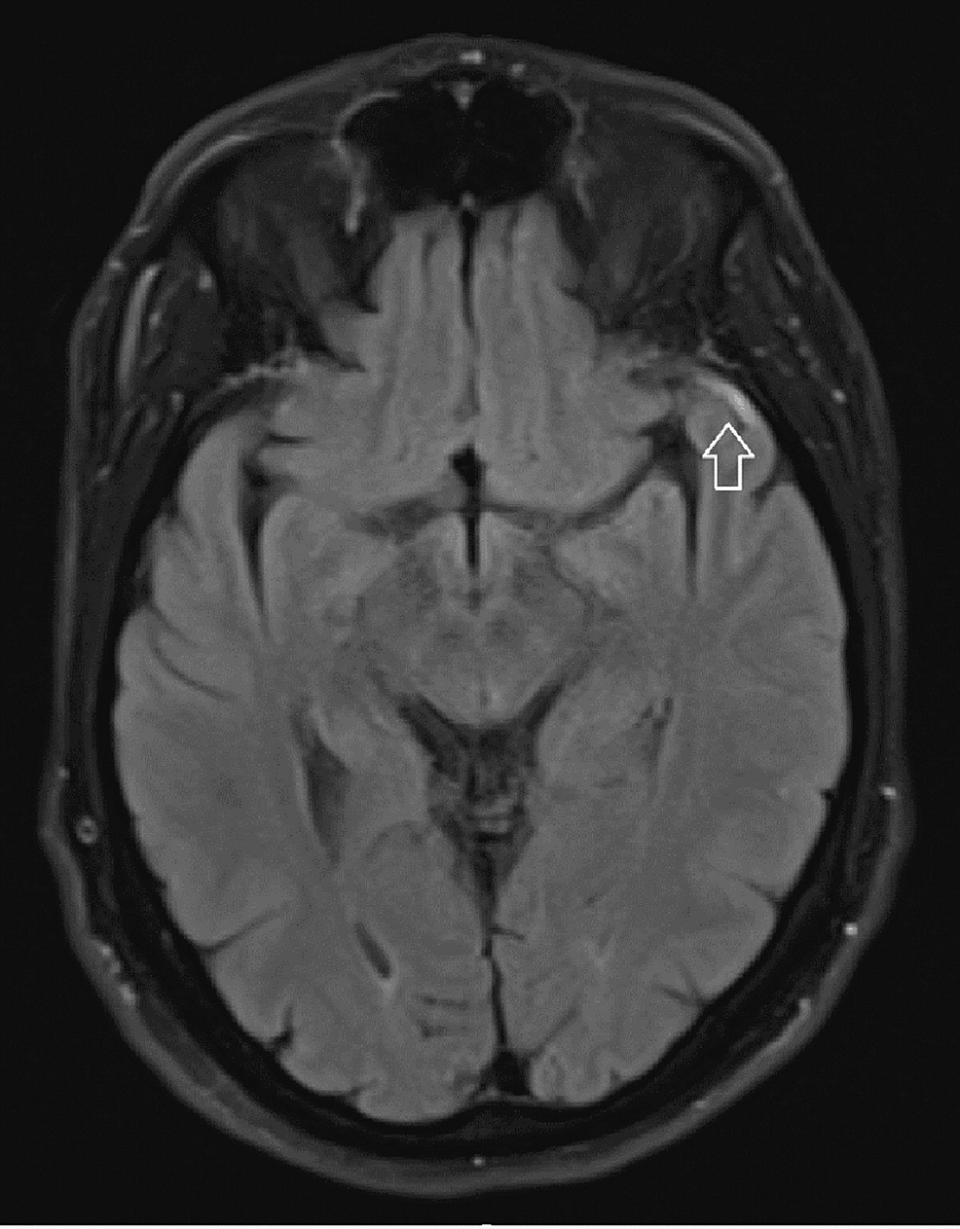
MRI-FLAIR (axial view) with hyperintensity signal due to thrombosed cerebral vein at the temporal cortex convexity MRI: magnetic resonance imaging; FLAIR: fluid-attenuated inversion recovery

**Figure 3:**
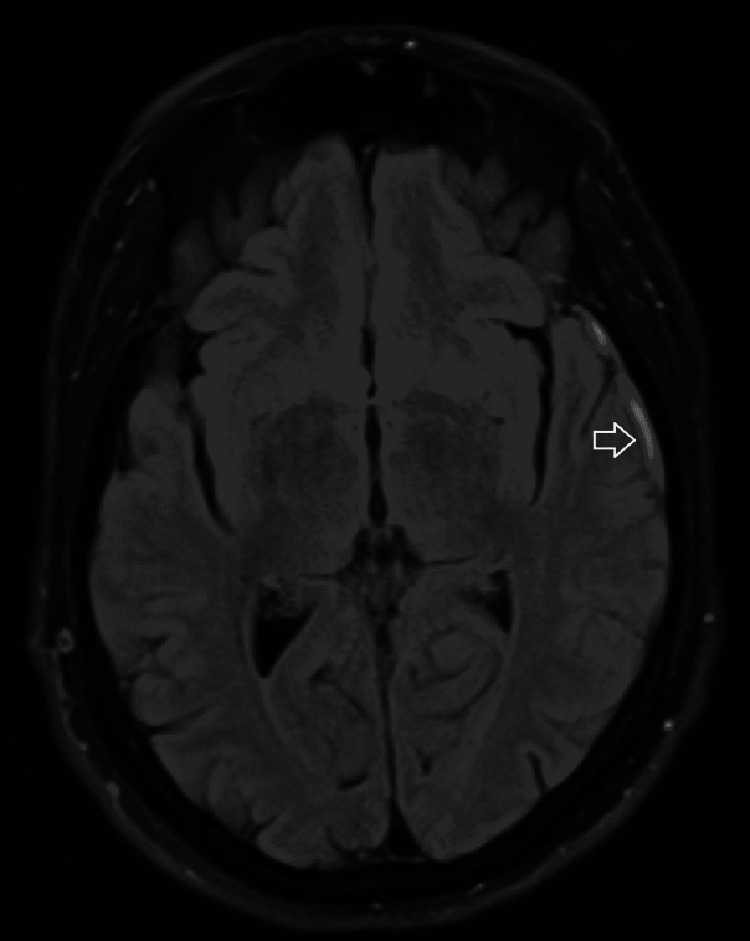
MRI-DWI showing increased signal at the cerebral vein thrombosis (arrow) MRI: magnetic resonance imaging; DWI: diffusion-weighted imaging

His brain magnetic resonance imaging (MRI) revealed superficial cortical venous thrombosis along the left cerebral convexity. We saw no focal diffusion restriction signal abnormality to suggest acute ischemic infarct. There was no evidence of recent bleeding or intracranial mass effect and no definite thrombosis of the superior sagittal sinus (Figures 1, 2, 3).

**Figure 4:**
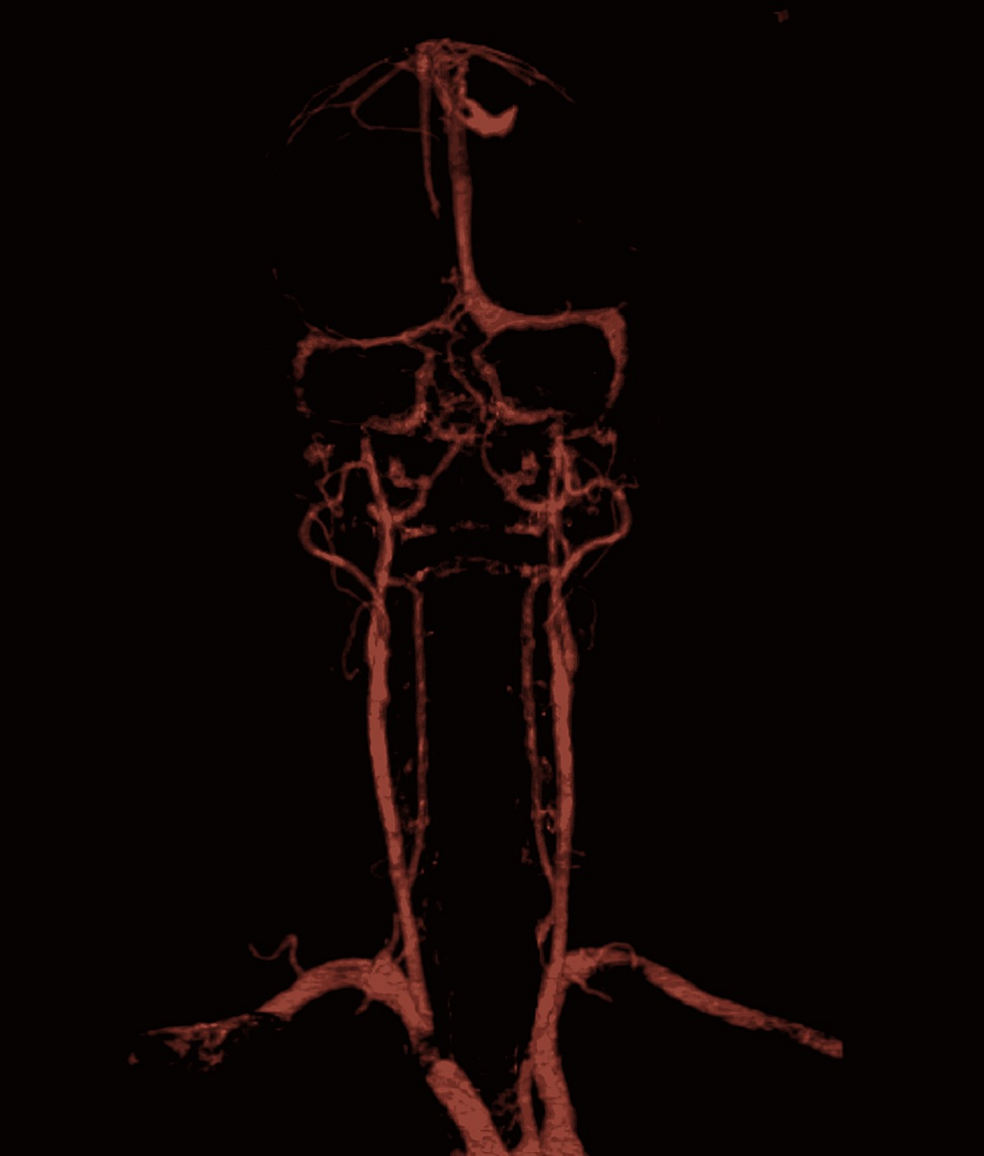
CTA of the next showing no large vessel (intracranial or extracranial) occlusion. No hemodynamically significant arterial stenosis CTA: computed tomography angiography

A computed tomography angiography (CTA) of his brain revealed no significant arterial stenosis; however, we noted acute thrombosis of the left superficial middle cerebral vein and a superficial cortical vein with anastomosis that drains into the superior sagittal sinus. The thrombosis terminated just proximal to the superior sagittal sinus. Proximal distended non-thrombosed veins were likely congested from increased venous backpressure (Figure 4).

**Figure 5:**
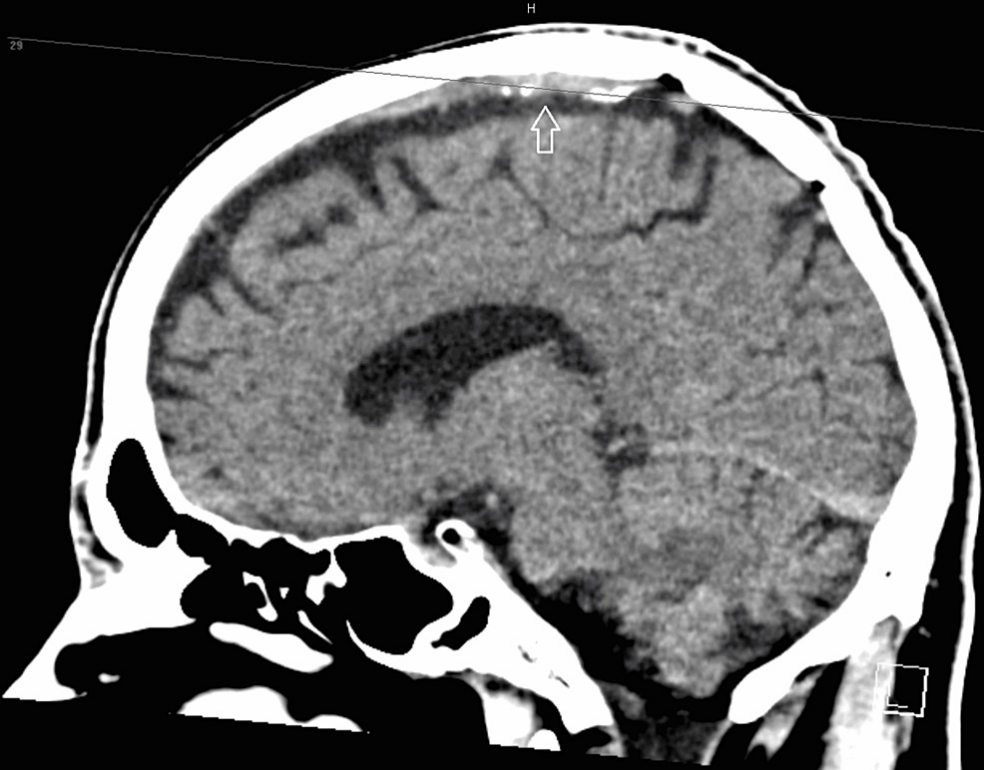
Repeated brain CT scan (sagittal view) showing no sign of bleeding or obvious infarction. Hyperdensity (arrow) due to thrombosed vain at the cortical convexity. CT: computed tomography.

During the first three days of his hospitalization, he had recurrent right-side upper extremity weakness, numbness, dysarthria, and headache that lasted for a few minutes to less than one hour. A repeated CT scan and brain MRI showed no new pathology (Figure 5), no bleeding or extension of the mentioned pathology, or new events. Between the attacks, he was completely normal except for the mild numbness that lasted up to six hours longer than the weakness. In one episode, he continued to have right upper extremity shaking and abnormal movement without loss of consciousness. We suspected focal seizure, and he commenced levetiracetam. On admission and because of his recurrent attacks, he started on intravenous unfractionated heparin, bridged with warfarin (target international normalized ratio of 2.5), aspirin, and levetiracetam.

**Figure 6:**
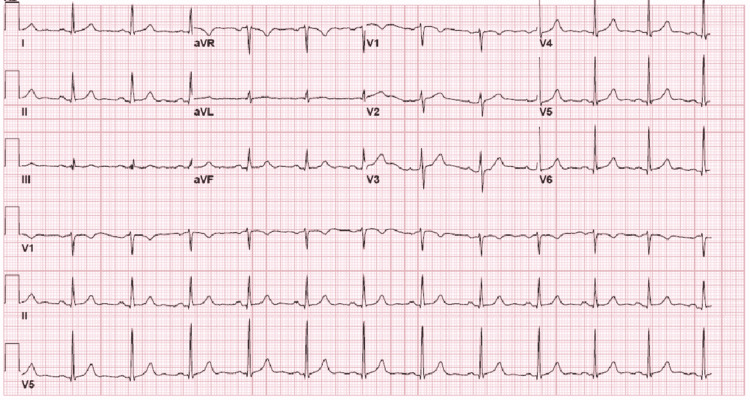
Standard 12-lead ECG showing sinus rhythm with no specific ST-T changes, no signs of RV strains, and no S1Q3T3 ECG: electrocardiogram; RV: right ventricular

**Figure 7:**
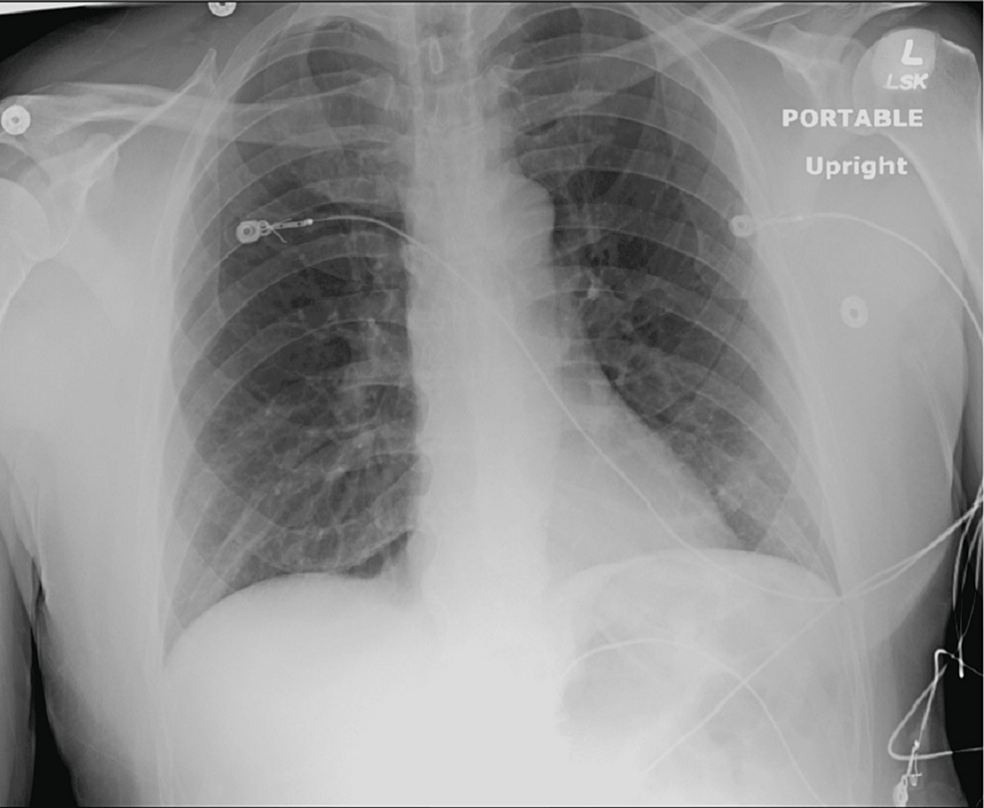
Chest x-ray showing no lymphadenopathy or obvious cardiopulmonary pathology

His workup for polycythemia was negative (no *JAK2* gene mutation), and his erythropoietin level was within the reference range. After hydration, a second complete blood count (CBC) revealed his hemoglobin was 15 mg/dL. His thrombophilia workup results were negative (no prothrombin gene mutation). His antinuclear antibody panel was 1:160 but negative for subtypes. His rheumatoid factor test was negative, and his c-reactive protein and erythrocyte sedimentation rate were within the reference range. His echocardiography and transthoracic echocardiogram results were unremarkable, and his electrocardiogram showed sinus rhythm (Figure 6). The patient's chest x-ray showed no lymphadenopathy and no cardiopulmonary pathology (Figure 7).

He was observed for two weeks and experienced significant improvement in his symptoms without recurrence of the mentioned episodes. He was discharged on direct oral anticoagulation (DOAC) medications aspirin, levetiracetam, and statin. He left the hospital before we could do a blood workup for B12, homocysteine, methylmalonic acid, double-stranded deoxyribonucleic acid, perinuclear antineutrophil cytoplasmic antibodies (ANCA), cytoplasmic ANCA, anti-Smith antibodies, ribonucleoprotein antibodies, and anti-histone antibodies. The patient had denied any clinical findings of systemic lupus erythematosus (SLE) or family history of SLE or thrombophilia. He also reported he was not taking any medications or herbals.

## Discussion

CVT is less common than arterial stroke, and presentation depends on which vein or sinus is occluded. However, CVT mimics arterial stroke in symptoms and signs, with unique signs related to location. An extension to the jugular bulb may cause jugular foramen syndrome, normal pressure hydrocephalus, cranial nerve injury, brain edema, or focal seizure. Herniation and brain death is rare. A magnetic resonance venogram is the best diagnostic modality. In most cases, a full workup with blood chemistry, coagulation profile, platelet count, and CBC is mandatory; screening every case for hypercoagulable status is recommended by the American Heart Association (AHA) and the American Stroke Association (ASA). Testing for protein C, protein S, factor V Leiden, antiphospholipid, prothrombin mutation, and antithrombin is recommended for diagnosis, and the results will guide the type and duration of therapy [7-10].

CVT has a variety of causes, such as trauma or during surgery with manipulation of the dura mater, sinuses, or cerebral veins during procedures, or lumbar puncture, thrombosis may result with or without cerebrospinal fluid leak. These can cause intracranial hypotension that leads to a decrease in mean blood flow, which can cause venous stagnation and thrombosis. CVT can also be caused by medications that induce a state of hypercoagulability. Such medications include steroids, erythropoietin, contraception, and tamoxifen. Several conditions can also lead to a hypercoagulable state such as pregnancy, inflammatory bowel disease, sarcoidosis, liver cirrhosis, thrombotic thrombocytopenic purpura, sickle cell disease, polycythemia, paroxysmal nocturnal hemoglobinuria, vasculitis, hyperhomocysteinemia, and nephrotic syndrome are increasing the risk of CVT [2,3,7,8,11]. Other risk factors for hypercoagulability include dehydration and high altitudes [2-4] and SARS-CoV-2 infection or a COVID-19 vaccination [5,6].

The cause of our patient's hypercoagulable state was multifactorial: our patient was always traveling, which puts him at risk of both dehydration and high-altitude exposure. His recent vaccination might have contributed to increasing the risk. The mechanisms of the vaccine induce thrombosis in several ways, through direct activation of inflammation, inducing thrombocytopenia, anti-platelet factor 4, or thrombotic thrombocytopenia [4-6]. It can also activate the coagulation cascade and the so-called syndrome of vaccine-induced immune thrombotic thrombocytopenic, which can cause arterial thrombosis [5,6]. The following table summarizes the articles that mention COVID-19 infection and vaccinations-related venous thrombosis.

**Table 1:** Literature review: summary of COVID-19-related venous thrombosis VITT: vaccine-induced immune thrombotic thrombocytopenia; IVIG: intravenous Immunoglobulin; TTP: thrombotic thrombocytopenia; PF4: platelet factor 4; SIRS: systemic inflammatory response syndrome

Author, Year	Results	Recommendation and summary
Gómez-Mesa et al., 2021 [4]	Two factors responsible for development of TTP during COVID infection are 1. Direct or indirect stimulation of SIRS due to cytokine storm, 2. Drug-Drug interaction	Encourage the use of anticoagulant prophylaxis during hospitalization for COVID 19.
Greinacher et al., 2021 [5]	Evaluated 11 patients cases who developed TTP after vaccination with ChAdOx1 nCov-19	Incidence occurs 5 to 20 days after vaccination. Post vaccine TTP is a rare complication related to platelet-activating antibodies against PF4.
Siegler et al., 2021 [6]	Evaluated 17 cases after Ad26.COV2.S vaccine and 169 cases after ChAdOx1 recipients developed VITT	Symptoms Onset 4 weeks after the vaccine. Low threshold for testing anti-PF4. Early intervention with IVIG or Plasma Exchange. The benefit of the vaccine overweight the risks, still it is reasonable to recommend an alternative vaccine if available.
Al-Mayhani et al., 2021 [12]	Three patients with VITT presented with ischemic stroke following the ChAdOx1 nCoV-19 Vaccine	High suspicious level at any neurologic signs or symptoms after vaccination with adenovirus-based vaccine. Work up looking for associated arterial or venous thrombosis, anti-PF4. Urgent intervention with IVIG, plasma exchange.

CVT treatment is divided into two phases: acute and long-term. During the acute phase, unfractionated or low-molecular-weight heparin is recommended. Surgical intervention or endovascular treatment is a more aggressive measure than medication and is only indicated for minor cases after the failure of medical management. For long-term treatment, vitamin K antagonists or direct oral anticoagulants (DOACs) are acceptable [9,10,11]. The duration of therapy is very similar to deep vein thrombosis treatment length. Therapy usually lasts three to six months for provoked CVT and six months to one year for unprovoked CVT. Lifelong treatment is recommended for patients with a high risk for venous embolism thrombosis or patients with severe thrombophilia, recurrent CVT, or venous thromboembolism. For such patients, DOACs or vitamin K antagonists are viable treatment options [8-17].

Our patient's condition was multifactorial likely related to frequent air travel, the COVID-19 vaccine, and dehydration. Therefore, we recommend advising appropriate patients to avoid flying sooner than three months after the vaccine, stay well-hydrated to avoid increased blood viscosity, and avoid frequent flights in the short term for people at high risk. The AHA and ASA encourage people to avoid flights after CVT for two weeks.

## Conclusions

Our patient represents a rare case of CVT in a middle-aged man, related to recent COVID-19 vaccination, traveling, and dehydration. Our case highlights that close monitoring of neurologic symptoms and signs is vital to prevent life-threatening complications including cerebral herniation, hemorrhagic transformation, and the associated morbidity and mortality. A thorough literature review showed that adenovirus-based vaccines were associated with a higher chance of VITT compared to others. The benefits of the COVID-19 vaccination outweigh the risks, still, it is reasonable to recommend an alternative vaccine for high-risk patients if available.
